# Materials and Places for Learning: Experiences of Doctoral Students in and around University Spaces

**DOI:** 10.1007/s42438-022-00328-x

**Published:** 2022-07-25

**Authors:** Lucila Carvalho, Cristina Garduño Freeman

**Affiliations:** 1 Equity Through Education Research Centre, Institute of Education, College of Humanities & Social Sciences, Massey University, Auckland, New Zealand; 2 School of Built Environment, Faculty of Arts, Design and Architecture, University of New South Wales, Kensington, Australia

**Keywords:** Learning spaces, Doctoral students, Sociomateriality, Belonging, Visual methods

## Abstract

People are more likely to thrive when they feel connected, when they feel they belong to a group, to a place, or when they feel part of a community. Places can play a powerful role in shaping one’s attachment to others and to institutions as part of the development of one’s identity. People’s experiences of places are linked to their sensorial impressions of material and digital elements, and to their perceptions of how multiple elements interconnect and impact lived experiences or imagined futures. This research investigates doctoral students’ experiences of places for learning in and around a university in New Zealand. The analysis combines individual interviews and digital multimodal artefacts created by participants who were studying on campus or studying at distance and remotely located. By acknowledging the diversity of university spaces where learning activity may unfold — in classrooms, at libraries, in the canteen, in the corridors, via online learning management systems, social media and messaging, or in the many in-between spaces such as buses, cafes, or working from home — this paper discusses the connections between people, places, material, and digital artefacts, with a focus on the materiality of learning in and around university spaces. Using socio-material conceptual lenses, the article reveals how students navigate the postdigital university through the spaces they inhabit and the places they value, and how their attachment to materials, feelings of inclusion, and learning purpose interconnect to support their (emerging) professional identity.

## Introduction

Covid-19 brought about a range of educational challenges, including the need to understand the evolving relationship between digital technologies and the learning spaces of higher education. Recently, Lamb and colleagues (2022) have called for a nuanced understanding of how we approach the concept of *postdigital learning spaces in higher education*, arguing that the term often accommodates a range of interpretations, in research circles and in everyday use, in conversations in and around universities. Creswell (2005) suggests that when speaking of *places*, one is evoking a relationship between humans and spatial configurations, that is, alluding to both something concrete and experienced, whilst *spaces* have a more abstract, figurative, and conceptual meaning. Accordingly, this research refers to *places for learning* to draw attention to the entanglement between students’ sense of intimacy, their emerging professional identity, and the blurred boundaries between social and spatial, material and virtual spaces. When referring to places for learning, we recognise students’ co-presence in multiple spaces through the notion of the postdigital, where the digital and physical spaces are framed as intertwined (Fawns 2019).

Places often evoke experiences that are entwined with belonging (Lovel 1998). A sense of belonging has been linked to individual growth and is deeply relevant for people’s mental health, their sense of self and identity (Peltonen et al. 2017). The relationship between place and belonging can be seen as shaping or reflecting people’s attachment to a community, through embodied experiences in physical spaces, as well as those imagined regardless of whether they are in the past, present or future (Altman and Low 1992; Blunt 2007; Cresswell 2012; Holton and Finn 2018; Massey 2005). This research investigates how places, on campus, at home and online interconnect to students’ sense of learning purpose and belonging, by focusing on a particular group of university students — doctoral candidates enrolled in a multi-campus university, which has provision for both on-campus and distance learning.

Many universities are represented, even defined, by their physical campus (Edwards 2013). Consider the ‘sandstone’ university, the city campus or even the ‘college town’ where buildings and facilities are dispersed. Universities are also institutions. They are shaped by specific social structures and bounded by hierarchies (Carvalho et al. 2018; Connell 2019). Bayne and colleagues (2020: xxix) suggest that students tend to see the physical campus and learning in these institutional places as a ‘guarantor of the authenticity of academic experience’ but warn that one should ‘not succumb to campus envy’. Indeed, students often conflate the university as an institution with its physical place. Nevertheless, a university involves physical and digital spaces *as well as* social structures.

Social structures within the specific context of a doctoral degree can be related to students being in the ‘in-between’ as they often aspire to become members of the academic staff (Mimirinis and Ahlberg 2021), some of them performing tutor roles whilst studying. These doctoral students might have different privileges (such as access to the staff lunchroom) in comparison with undergraduate students who are looking towards establishing careers elsewhere. As doctoral students embark on the processes of becoming (e.g., a scholar, a professional, a researcher and so on), many are aspiring to embrace and embody a new identity as an academic expert in a particular field (Barnacle and Mewburn 2010; Mantai 2017), which requires deep commitment and will power. According to Alexander and colleagues (2014: 172) ‘the doctoral trajectory can be a solitary activity accompanied by uncertainty and a temporary sense of displacement’. Doctoral candidates have to work hard to acquire sophisticated skills, whilst displaying a strong self-driven nature to focus and engage in research activity for extended periods of time. This can be a challenging experience where the doctoral student is becoming an academic colleague.

Universities offer academic and social support to doctoral students through several non-compulsory activities throughout their candidature time, delivered within a range of places. These may include practical guidance and access to material tools and resources, such as when a doctoral student has a dedicated physical space for studying on-campus, like an allocated desk and room. They may also have access to digital tools (e.g. laptops, audio-recorders) and resources such as the library collection, specific software for research and/or knowledge databases. Doctoral students may be supported through academic opportunities specifically oriented towards developing their research skills, via in person and online workshops, events or practical initiatives, such as the financial support provided by scholarships. While these institutionally driven activities are important, many invaluable opportunities for learning occur informally, through participation in social activities; for example, when doctoral students wander through the in-between, non-teaching spaces of a campus (Boys 2011) — the university cafes and canteen, the corridors, staff rooms, foyers of lecture theatres or other informal spaces where students, professional staff and academics might happen to meet. Similarly, within the digital realm, doctoral students also often interact with the university as both staff and student, navigating both identities on formal platforms, such as a learning management system, when applying for ethics or more simply, the university’s website. While these sites may function as places for students to reach out for academic support and information, they may also be used in combination with other less formal or informal digital opportunities, such as those afforded by messaging apps (e.g. WhatsApp groups) or social media (e.g. a Twitter account of a university or a self-managed Facebook group for doctoral students).

Drawing on the concept of *place attachment* (Scannell and Gifford 2010; Smith 2019; Diener and Hagen 2022), this paper discusses the interplay between the qualities of physical and digital materials and how they influence students’ learning activity within an ecology of elements (Carvalho and Goodyear 2014; Carvalho et al. 2017). This interplay is explored within the multiple spaces doctoral students inhabit and the places they value. Using selected accounts from students describing their attachment to the university, this article discusses place attachment as connected to feelings of inclusion and social orientation, grounding students’ learning purpose or supporting their (emerging) professional identity. The next section, introduces the theoretical framing of this research, including the socio-materialist lenses that support the theorizing of the materiality and spatial configurations of universities. Next, a review of recent literature on learning spaces is presented, as well as literature examining spatial and social experiences of doctoral students. Finally, the research study is discussed: its participants — doctoral students learning on-campus and at distance, the analytical and visual methods employed and the themes that emerged around the way in which places and materialities afford them a sense of belonging and contribute to their emerging professional identity.

## Place Attachment and Materiality

Work on space, place and materiality has been conceptually developed in several fields, including geography, architecture, anthropology, sociology and education, with the term *place attachment* being used to express its social and physical nature (Smith 2019). Social scientists and psychologists might speak of social places whilst characterizing degrees of bonding and refer to physical places as connected to one’s rootedness (Scannell and Gifford 2010). In contrast, geographers might focus on what makes each place unique, or on how a place connects to meaning in the lives of people who are in a certain location (Smith 2019). For Diener and Hagen (2022), place attachment often foregrounds people’s emotional connections to places and is related to how those connections shape thought and behaviour. A theoretical framework by Scannell and Gifford’s (2010) offers a way of conceptualizing place attachment by focusing on the ‘person’ (or on *who* is attached and why), the ‘place’ (or on *what* people are attached to) and the ‘process’ (or on *how* a place becomes meaningful to someone and its impact on individuals). In this research, our focus is on the ways doctoral students personalize and customize spaces to make these their own, to create a sense of belonging and attachment during their doctoral candidature. As such, the notion of place attachment is used here to refer to the emotional, symbolic and affective dimensions of one’s connections to places for learning (Altman and Low 1992; Rishbeth and Powell 2013). It involves understanding the ways students might turn a *space* into their *place* for learning, whilst reflecting on their attachment to the symbolic nature and the materials and spaces of a university.

Within the context of this research, we consider that the built environment of a campus has a material and a symbolic relevance for all students, including those who attend the campus and those learning ‘at the university’ whilst remotely located (Bayne et al. 2020). As an institution, universities can be seen as a community — a place where one belongs and draws identity from (Holton 2015; Holton and Riley 2014; Yu et al. 2018). Students often attend university during a limited period of years, but for some, this connection is reflected within their identity, as they become professionals, or alumni. This relationship is showcased through social media and professional networks: ‘I am a graduate of X university’. The efforts and spending on university branding, marketing, ranking and corporatization contribute to reinforcing such connections between institutional branding and long-term student and alumni identity.

For universities with multiple campuses and those operating simultaneously via different modes (on-campus and at a distance), an extra challenge exists in making sure the university is seen as a singular coherent institution. Bayne et al. (2014) note that the experiences of university students learning via distance modes challenge traditional views of what it means to be at the university. As the authors examined the symbolic and material attachment of distance students to the physical campus, they observed that students, even those who may have never physically attended the campus, often create their own versions of the spatial configurations of a bounded campus space. Distance students mix specific ways of being part of the university (enabled by online connections and digital representations of the university) to other types of spatial connections, which reflect their stories about what it means for them to be ‘at the university’. As such, distance students’ symbolic constructions of the physical campus can often be mediated through material and digital elements and formal and informal online representations, including university’s brochures, websites, social media sites and so on.

Socio-materialist conceptual frameworks enable a deeper understanding of the relations between the social and material, cultural and technological aspects of doctoral students’ experiences. A ‘turn to matter’ has been observed in many studies in the arts, humanities and social sciences, in the research of organizational theorists, anthropologists and others (see Barad 2007; Fox and Alldred 2015; Orlikowski 2010). Materialist approaches encourage us to shift the focus of a social inquiry, from perspectives that foreground humans and their bodies, to instead emphasize ‘how relational networks or assemblages of animate and inanimate affect and are affected’ (Fox and Alldred 2015: 399). Socio-materialism allows us to focus on the materiality of learning but without losing sight of their entanglement with humans, within a wide web of elements (Fenwick et al. 2011; Fenwick 2015; Sørensen 2009). Sørensen’s (2009) notion of patterns of relations highlights that humans and non-humans are both entities of a network, where the main focus is on understanding connections and the dynamic practices that emerge. These patterns of relations highlight the web of elements involved in the carrying out of everyday activity, going beyond techno or human-centred accounts, towards exploring wider links between multiple elements. Relations between elements are seen as enmeshed in people’s everyday practices and connected to particular ways that things and humans may act on one another. Through acting on one another, things and humans mutually influence and transform the characteristics of the other and the activity that emerges. Indeed, an interconnectedness of elements can be seen in all sorts of learning scenarios. For example, in the physical environment of a ‘face-to-face’ classroom, students may be using a laptop and the Internet to research a given topic whilst still co-present with lecturers and peers in the physical space. Or students and lecturers may be participating in a conference call whilst also physically bounded to the places their bodies are located. One may be attending an ‘online class’ using a smartphone while travelling on a bus, seating on a café or at the library. These types of scenarios raise questions about the boundaries of places for learning, making it hard to distinguish where the ‘face-to-face learning’ transitions into ‘online learning’; where the material and digital intersect (Gourlay 2021). Indeed, recent debates in education have been critical of descriptions of learning activity that are detached from the physical realm (they never are), challenging notions that see students and lecturers as disembodied and displaced (Bayne and Jandrić 2017; Gourlay 2021; Gourlay and Oliver 2018; Fawns 2019). This new state of co-presence in multiple spaces is foregrounded through the postdigital, as we review recent research that builds on this entwinement within the context of learning spaces of higher education.

## Learning Spaces

Learning spaces have been the focus of many studies at the intersection of architecture and pedagogy, with researchers examining relationships between built forms and human activity in higher education, including learning activity beyond formal classroom settings and those often connected to serendipitous encounters (see Boddington and Boys 2011; Boys 2011; Oblinger 2006). Indeed, learning spaces in higher education have been described as fluid, challenging traditional notions associated with images of formal classrooms, such as lecture theatres or libraries (Lamb 2019). These types of formal spaces often account for only part of a picture and should be seen in combination with other types of spaces for learning, such as social spaces (e.g. the university café, or canteen), the transitory spaces (e.g. a train ride to the university, the university corridors) or those in our domestic settings (e.g. a home office, the kitchen table) where students often also engage in some sort of learning activity. Furthermore, the notion of learning spaces can be associated with the ‘virtual’ realm, when involving the use of digital technologies, in discussion boards and learning management systems, synchronous sessions via conference calls or when students interact with other students through social media platforms (Oblinger 2006). Gourlay (2021: 57) notes that whilst dominant debates in education recognize a shift in the nature of learning spaces, many discourses still foreground the digital as ‘a disembodied realm, outside of the material world’. In exploring the nature of space, absence and presence, Gourlay (2022) suggests social topologies and the notion of fire space, discussing simultaneous absence and presence, being ‘there’ and ‘not there’ and a more nuanced understanding of interactions in ‘online’ education. Lamb (2019: 23) also reminds us that it is important that the digital is understood as ‘entangled with the physical settings in which they are accessed, in conjunction with a wider range of human and material resources’. And as Massey (2005) suggests, spaces are constantly evolving and always under construction.

The multiple ways learning spaces can be studied and managed, the ways form and function subtly impact learning and what indeed constitutes a learning space in postdigital higher education have been problematized on a recent special issue edited by Lamb and colleagues (2022). An interesting contribution by Boys (2022) calls for a critical debate about learning spaces and inequalities in access and inclusion, for noticing how certain forms of behaviours and spaces contribute to some people being seen and valued, whilst others continue to be at the margins or are made invisible. Existing tensions and contradictions about what counts as appropriate behaviours and settings for teachers and students became even more widely apparent during the pandemic, revealing unconscious biases in higher education. As Boys (2022: 32) argues, such inequalities will only be addressed when we are able ‘to see how the encounters, space, time and technologies of higher education are already differentially distributed and how underlying discriminatory patterns are currently being ignored, challenged or reframed’. A critical stance is also foregrounded by Goodyear (2022), in a discussion of learning spaces grounded on design justice, educational infrastructure and social innovation. As Goodyear (2022: 52) remarks, ‘creating new spaces for learning requires us to work (reflexively)’ to develop capacity that allows ‘people to aspire, to look up, to look at the future and map plausible routes towards states of affairs that they have reason to value’. Foundational to this discussion is to identify the high-level values and ways of working that will help us realize the university that we desire. Overall, these recent postdigital studies invite us to consider learning spaces beyond their physical dimensions and/or teaching and learning practices; they invite us to carefully notice the complex configurations of human and non-human actors, and how this assemblage extends far beyond single physical elements or pedagogical practices, towards university strategy, government policy, digital technologies and so on (Lamb et al. 2022). And as Spire (2022) suggests, they reveal that there are many tensions, such as those related to the influence of materials in the socio-spatial environment of universities, which call for a better understanding of *whose* aims and objectives are at stake and *how* certain values and principles might be expressed through the material and socio-spatial environments of universities (Boys 2022; Goodyear 2022; Spire 2022). In line with these ideas, in our research, we draw on a notion of learning spaces that foregrounds people, places, material and digital elements and the materiality of learning in and around university spaces — being these at classrooms, at libraries, in the canteen, in the corridors, via learning management systems, social media and in the many in-between spaces students and lecturers navigate, including when working from home. In the next section, we look more closely at literature exploring spatial and social experiences of doctoral students.

## Spatial and Social Experiences of Doctoral Students

The experience of joining a university has been related to experiences of entering a new community (Holton 2015). Students embrace a new identity, values and challenges, whilst learning new knowledge and novel practices. The social experiences of doctoral students are often grounded on a range of privileges and restrictions connected to the hierarchical social structures of academia. In comparison with undergraduate students, doctoral candidates may be privileged in having access to allocated office spaces, staff facilities and resources — like stationery, computers, printers, and the staff tearoom. And yet, these students are not often recognized as staff members or as ‘true academics’ (McAlpine et al. 2014) — they are excluded from staff meetings and social functions residing mostly at the bottom of the hierarchical academic structure. Despite their ‘student status’, doctoral candidates might well be mature students returning to education to embark on a new adventure, after having had established jobs and careers. Some might be under some financial burden or have carer’s responsibilities commensurate with their stage of life. These conditions mean some might experience feelings of isolation; some doctoral students might need institutional support, or experience additional disadvantages, particularly those amongst minority groups (Mattocks and Briscoe-Palmer 2016). Doctoral students also report having an ambiguous sense of self, which brings questions about belonging and learning purpose to the fore, especially in reference to social norms and spaces within the university (Phelps 2016). Mantai (2019) argues that in addition to academic and financial support, doctoral students need strong social connections to develop and establish their identities as researchers. These social connections not only benefit students’ physical and emotional well-being, but they also encourage progress, and are seen as an investment in a researcher’s career (Peltonen et al. 2017). Mantai and Dowling (2015) highlight the need for diverse types of social support, such as moral, emotional, guiding and mentoring, as well as companionship and collegiality. Such studies reinforce the importance of peer learning and academic networking during doctoral studies, and the valuable lessons more advanced students may share with those at early stages of their journeys. Having a network of like-minded others, who are undergoing similar experiences, can help doctoral students overcome their loneliness (Deem and Brehony 2000; Meschitti 2019). Even though the main task of a doctoral degree is about making an original contribution to a body of knowledge — researchers have noted that a successful academic career involves also learning how to participate effectively in social aspects of academic life (Mewburn and Thomson 2013).

Students attending the campus have perhaps more informal opportunities to engage in peer learning (Meschitti 2019), as they are often exposed to a range of academic practices, for example while sharing office spaces with other students, or by having access to rooms shared by academics. The built environment plays an important material role in facilitating such interactions and conversations, which were greatly missed during the recent lockdown restrictions of Covid-19. Barnacle and Mewburn (2010) suggest that specific spatial arrangements of the physical environment are likely to contribute to knowledge and identity formation of doctoral candidates in different ways. For example, the collocation between students and their proximity to academics (such as having access to the staff tearoom) implicitly speaks about the research environment, its social norms and hierarchies, which in turn impact on the candidates’ learning. This is in addition to other more formal opportunities, such as those afforded by workshops, seminars, presentations, supervision and so on.

Students who are remotely located are likely to be more heavily reliant on technology and online connections to access and maintain their networks than those located on campus. But as Bayne and colleagues (2020) remind us, the power of online interactions should not be underestimated, as they may also open new, creative, highly engaging ways of teaching and learning. They enable the building up of new proximities, which are different from what unfolds in a physical space, but they can also be highly positive and valuable. Importantly, the physical space of a university has a symbolic significance for all — those on-campus and those at a distance. Exploring doctoral students’ unique social, cultural and institutional experiences may help us understand how to best design for a more inclusive doctoral environment, one that reflects the voices and needs of all students and allows us to work towards building a learning community, or the university we desire (Goodyear 2022). The next section details the background for the research study, including the research design, followed by the analysis and discussion of emerging findings of this research, with a focus on place attachment, materiality, belonging and identity.

## Project Description

Von Benzon et al. (2021) call for more qualitative, ethnographic, participatory and experimental research methods — or for reflexive methods. Examples of innovative research using multimodal methods to analyse students’ experiences of learning spaces include Bayne and colleagues’ (2014) research on the social topologies of distance students, Gourlay and Oliver’s (2017) exploration of longitudinal multimodal journaling and the spaces and practices of postgraduate students, Lamb’s (2019) digital postcards in the study of learning spaces and practices and Wardak et al. (2022)’s digital stories created by students about their experiences of remote learning during lockdown. Building on some of these ideas and on our previous research that connects social values, community values and the built environment (Carvalho and Garduño Freeman 2016, 2018), this project explores doctoral students’ symbolic and material attachment to the university, with a focus on their experiences of place attachment, materiality, belonging and identity, using qualitative reflexive and creative methods. This included semi-structured interviews and the participants’ production of digital postcards — that is, digital artefacts comprising of visual imagery and audio files (Denzin and Lincoln 2017; Jewitt 2009; Price et al. 2013). But what is important here is not only the data collected, but *how* it is collected or produced. The use of creative methodologies has shown how making something — be it as complex as an artwork or as simple as a digital postcard — offers an iterative and reflective process for the participant (Gauntlett 2007, 2011). This means that the responses, rather than being only direct responses given under pressure in the space of an interview or survey, tend to be more nuanced and more considered as the participant has time to reflect on their contribution. Furthermore, the use of digital media, photographs in particular, has the advantage of being a ubiquitous form serving as both, a memory artefact and a message that can be analysed in terms of its content, its production and its reception (van House and Ames 2009). In this research, digital postcards identified and captured images of places for learning around the physical campus and at home locations, or of symbolic items, which were significant to the students themselves, and then gave them opportunity via the audio to reflect on these before contributing them to the project.

The overarching research questions that guided the study was: *How do materials and places for learning influence distance and on-campus students’ experience of place attachment, connection and belonging to the University?*

### Study Design, Participants and Data Collection

The study involved ten doctoral students in a multi-campus university in New Zealand, who were contacted and invited to participate in the project via the university mailing system and through face-to-face requests. The sample included on-campus students (Campus 1 *N* = 5, Campus 2 *N* = 2) and distance students (*N* = 3). Participants came from different disciplinary areas of research in the humanities and social sciences, in education (*N* = 5), sociology (*N* = 3), linguistics (*N* = 1) and defence studies (*N* = 1). All participants were over the age of 30 and had no direct relationships with the researchers.

There were three main phases in the study, which were sequentially conducted during the period of a year. In the first part of the project, eight students participated in an in-depth semi-structured individual interview (see Table 1) which allowed us to engage in in-depth conversations about the places students valued. Questions explored students’ favourite spaces, effective spaces for learning, usual spaces they used, spaces that they felt connected, social spaces, spaces that students identified with being a student at this particular university and so on. Interviews lasted between 30 minutes and 1 hour and were audio recorded and transcribed for analysis. Interviews with on-campus students were conducted at a private room/space in the university grounds, and interviews with distance students were conducted using conference call software.

**Table 1:** Participant distribution — interviews and digital postcards

	**Campus 1**	**Campus 2**	**Distance**
Part 1: Interviews	Participant 1 Participant 2 Participant 4	Participant 6 Participant 8	Participant 3 Participant 5 Participant 7
Part 2: Digital postcards	Participant 2 Participant 4 Participant 9 Participant 10		Participant 3 Participant 5 Participant 7

**Fig. 1:**
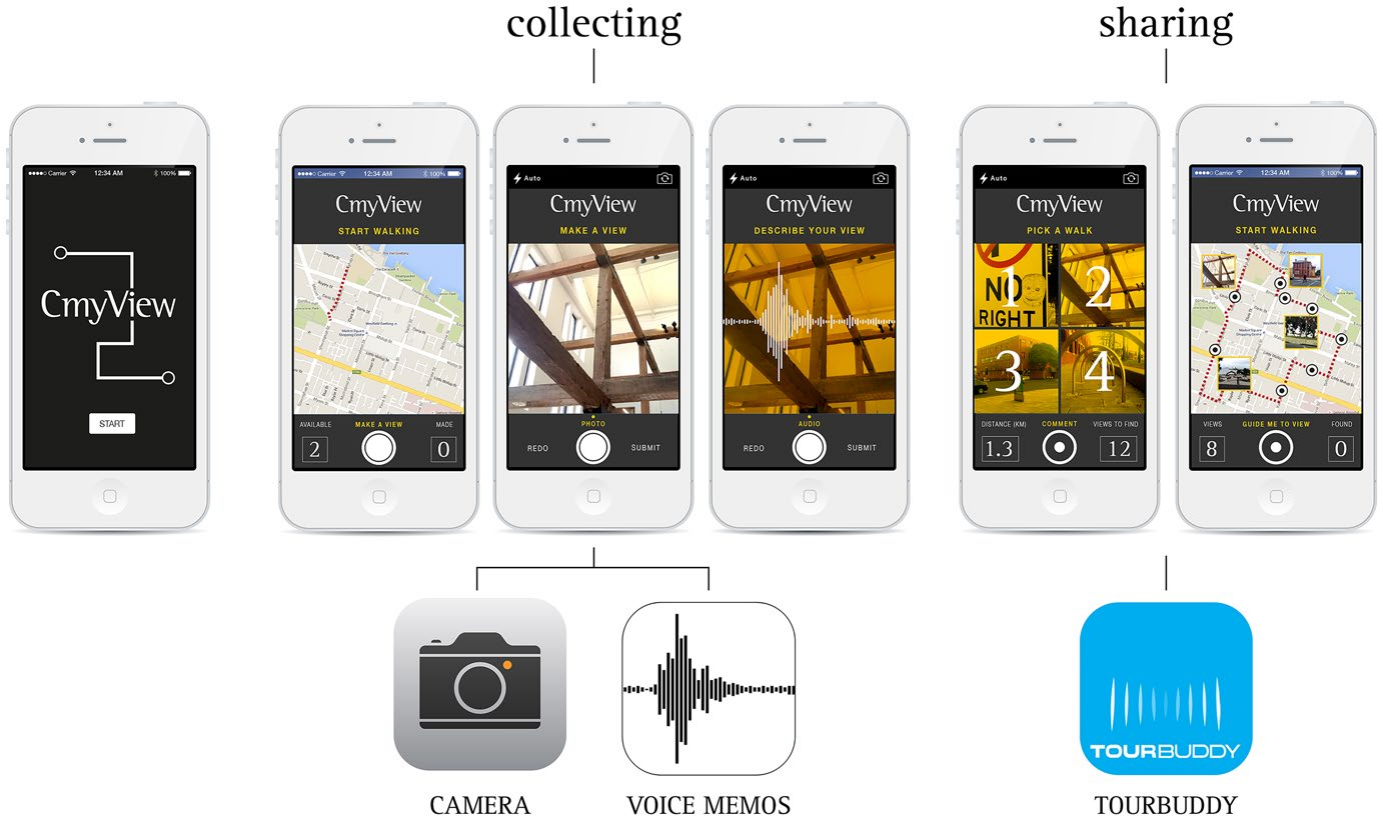
The CmyView app and a portfolio of digital postcards

In the second phase of the project, we drew on the CmyView methodology (Carvalho and Garduño Freeman 2016, 2018), asking students to create digital artefacts that contained walking trajectories around the campus and/or off campus. The CmyView methodology involves collecting and sharing views by making images and audio recordings of personally meaningful sites people see. Participants can collect these images while walking outdoors in the natural environment or indoors in the built environment. A person might collect 5–10 images to create a portfolio of digital postcards within a ‘walking trajectory’ (which contains images, audio and geolocation files), and this then becomes a traceable artefact, which can be shared with a community (Fig. 1). This methodology is based on existing mobile apps that focus specifically on mapping walks (e.g. Map my Walk, Glympse or Trails) and apps that encourage posting/sharing photographs (e.g. Instagram, Flickr, and Facebook), extending these by adding the ability to make an audio recording and linking it to the GPS point where the image was created. Our previous research employed the CmyView methodology with a group of undergraduate architecture students to explore networked heritage practices and community participation, when students documented their own and experienced others’ social values of the built environment (Carvalho and Garduño Freeman 2016, 2018). In the study described here, the artefacts created by students combined images of places for learning and short audio recordings in which students narrated why those places were of significance to their learning. Through the ‘making’ of the digital postcards, participants engage in an iterative and reflective process as they carefully ponder, select and collect images and then audio record their impressions (Gauntlett 2007, 2011). Their final productions capture representations of places and a message, where participants explain their values, feelings and reasons associated with choosing to represent those places. The digital postcards offer nuanced accounts which complement what can be captured through conversations in an interview. Digital artefacts created on campus were then curated into the CmyView app, to become traceable artefacts with multimodal elements (images, audio, video, route and GPS data) that encapsulated the experiences of on campus participants. These were then shared with other participants in the third phase of the project. The third part of the study involved students choosing a trajectory created by another student, or in other words, students ‘walked the walk’ created by other students whilst looking at the places where images were created and hearing their narrations of the places. In this article, we report on Phase 1 and 2 of the study.

Participants were asked to generate around 3–5 postcards. A total of 32 digital postcards were created by on-campus students and 8 digital postcards by distance students, using iPads or smartphones. On campus, students tended to create more postcards exceeding what was requested, for example, one participant created 9 digital postcards. Distance students kept their creations to two or three. The audio data of individual interviews and the digital postcards were transcribed for analysis. For brevity of presentation, findings reproduced here use transcriptions of selected audio passages of the digital postcards, reproduced alongside the digital images created and captured by the participants. Ethical clearance for this study was obtained from the Human Research Ethics Committee of the host university.

The qualitative nature of this research called for an interpretive approach (Denzin and Lincoln 2017), through thematic analysis that combined the interviews and multimodal artefacts (digital postcards) created by participants. The use of multimodal artefacts was important as it allowed participants to communicate their experiences through visual, aural, embodied and spatial interactions (mediated by the camera) with the environment (Jewitt 2009; Price et al. 2013). The analysis involved identifying passages in the interviews while visually analysing the digital postcards and thinking of the way the audio allowed the multiplicity of meanings that images can hold to be ‘pinned down’ (Chaplin 2006). The researchers looked for similar themes, which were then clustered together for analysis. The themes discussed in this article are those that students associated with places for learning that were significant to them, in and around their university experiences, reflecting students’ narratives of places, belonging and materials that seem to contribute to their identity and express their value on connections between nature and learning.

## Findings and Analysis

### Theme 1: My Place for Learning — Places that Represent Choice and Learning Purpose

For some on-campus participants, the value of having a physical space at the university is emphasized through narratives that reflect their use of materials to make the space fit their learning purposes. This is illustrated in Participant 6’s remarks, where she brings her experiences of studying or working in a dedicated space at the university, whilst comparing it with trying to be productive at home. Her narrative suggests a sense of choice and purpose, which is displayed in her experience of the space the university has allocated as ‘her productive place’. She reflects on a place for learning that allows her to focus, and on using materials (e.g. a notice at the door) to signal she is busy.

I think because, because work is a product of space for me so I found, if I try to work at home on my PhD I would be doing the dishes or hanging the washing out or you know there are a million things to procrastinate with whereas being at work in my office I am used to being productive there so I was productive on my PhD there. … I just had to set sort of boundaries around it or have that notice on my door saying I am working on my PhD so my door was shut, people mostly knew not to interrupt me. (on-campus 2, Participant 6).

Similarly, having a dedicated space to study is also highly valued by those who do not necessarily ever come to campus. Participant 5 refers to the value of a dedicated physical space, even though in this case the space is not associated with a university provision. There is a careful placement of things, perceived as supporting the particular ways she likes to work. A digital device (her laptop) is instrumental in allowing her to conduct her research, but she notes embodied aspects of her experience too, such as a space where there is no interruption and it is warm, and it is ‘the right kind of environment’:

I think place is important, having a place where you, you study and where you feel kind of safe from interruption and warm and kind of in the right environment is important but yeah it varies also depending on, on how long I’ve been studying … and depends on what I am doing … probably the most, that is related to the time when I study I do the best work very early in the morning so, which means I do most of my study in bed with the computer in, on my lap. (distance, Participant 5).

The research literature highlights the importance of the built environment in facilitating the connections or ‘the right state of mind’, of specific spatial qualities and arrangements that contribute to knowledge creation and identity formation of doctoral candidates (Barnacle and Mewburn 2010). Participant 2 values her place on campus, recurrently mentioning ‘my things’ in her account*.* Having a dedicated space helps her feel she belongs to the university, she has a place to call her own — this is ‘my space’. She talks about the physical feeling of ‘being a student’, as something strongly connected to ‘being on-campus’.

My favourite space is the PhD room, because I have my computer over there and I have my desk of course, and you know because is my thing I feel some sort of, a sense of also belonging to that space, which is mine … I’ve never thought about any other [way] of being a student. In my mind being a student is defined by being here physically … I don’t know why, but I never think about any other way of studying rather than doing it physically, maybe because I have enough time to come and attend to the space. (on-campus 1, Participant 2).

**Fig. 2:**
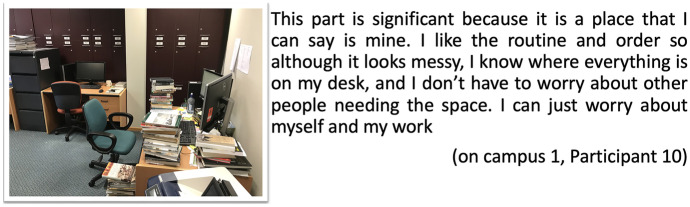
Postcard my desk, on-campus 1, Participant 10

**Fig. 3:**
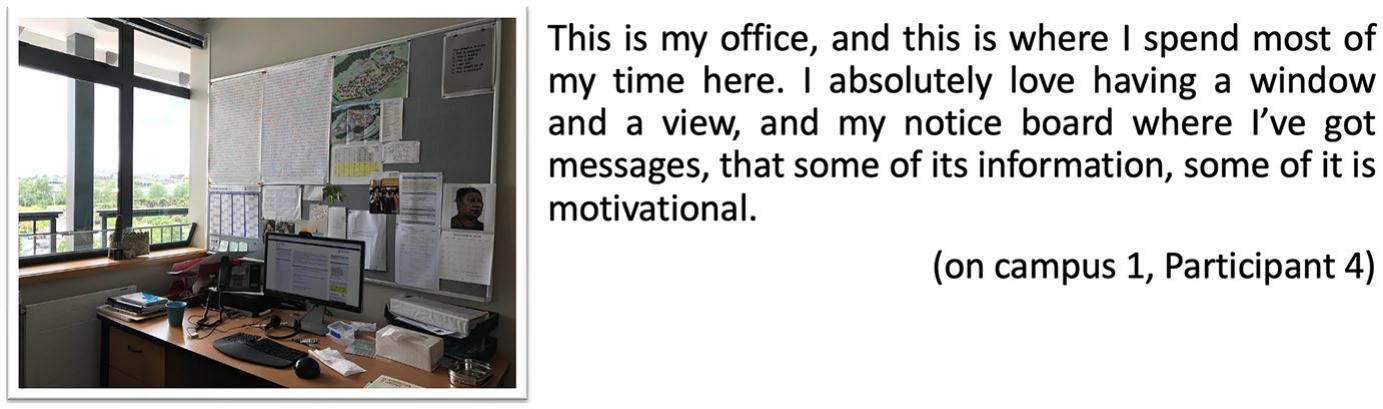
Postcard my room, on-campus 1, Participant 4

**Fig. 4:**
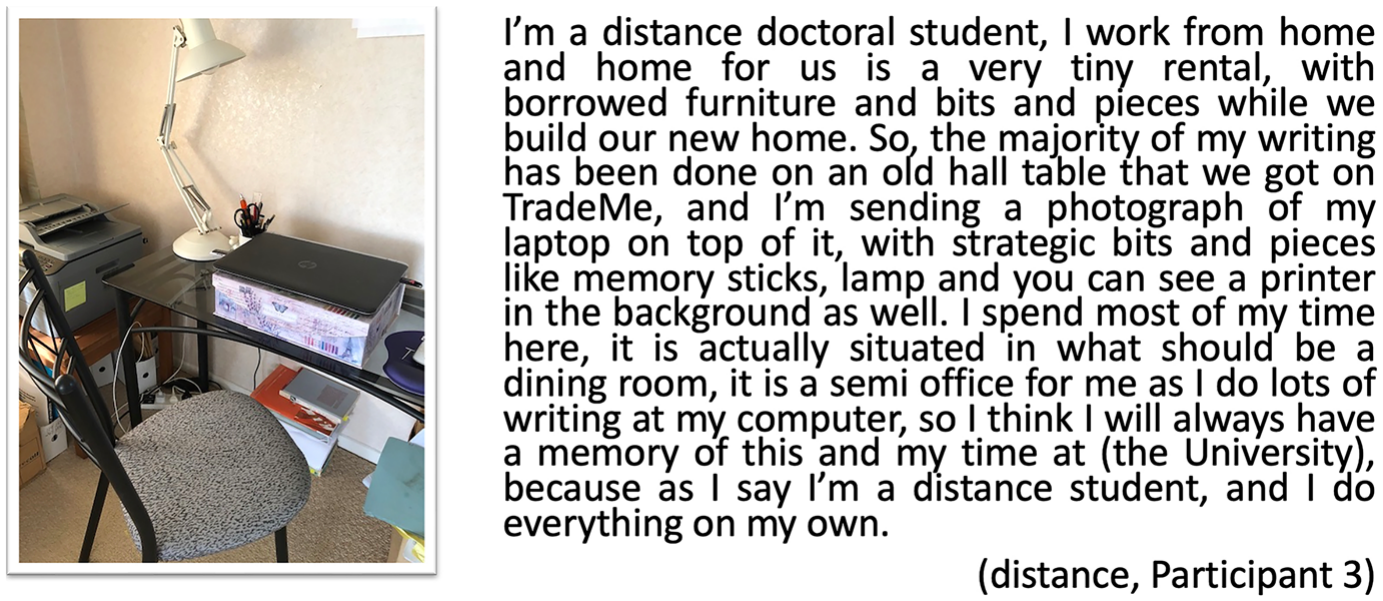
Postcard my desk, distance, Participant 3

Similarly, many digital postcards visually represented students’ desks, their books and surrounding materials, both for students on-campus and for those at distance. These items are ordinary, yet they have the capacity to enable students to feel a personal connection to the university and to their transforming identity as researchers. And arguably, it is not only the physical examples, but the personalisation of their desktop set-up, background, file systems, citation managers that too facilitate their emerging identity. As such, students’ experiences and interactions with materials and digital tools influence their goals and impact their sense of place through the choice of materials they keep around them, and the ways these in turn contribute to their sense of learning purpose. Like Participant 2 earlier, Participant 10 also refers to ‘a place that I can say is mine’ (Fig. 2). And like Participant 5, he also alludes to the careful placement of things, to support the ways he likes to work, and even though ‘it looks messy’, he knows ‘where everything is’:

Similarly, Participant 4 also talks about her things, the noticeboard, the messages in post-its positioned around her desk (Fig. 3). The location and things around her are important and help her keep motivated:

This idea of a dedicated place for learning is also seen in the digital postcards created by those who are not on-campus, reflecting the importance of materials around them. Participant 3 is a distance student, who rarely comes on-campus. She refers to the positioning of memory sticks, lamp, printer and other objects around her dedicated place for learning (Fig. 4). Her account connects to Bayne and colleagues’ (2014) descriptions of the new proximities of distance students, as Participant 3 uses the representation of her desk at home in explicit reference to her time ‘at the university’:

### Theme 2: Materials and Places that Help Shape Identity

Students also express the power of symbolic objects that represent their studies — such as the doctoral regalia. Participant 3 brings a story at the beginning of her doctoral journey, telling of a moment when she was attending a workshop, and students were invited to try on a doctoral hat. Students were asked to look at themselves in a mirror, and to take a picture. In reflecting on the sensory and embodied feeling of the object on her head, Participant 3 speaks about how this experience helped her envisage the end of her journey of becoming an academic, and how being asked to capture that moment as a permanent memory (a digital photo) has given her an opportunity to revisit and re-live this moment. Participant 3 talks about the ability to project the feeling (of accomplishment, of something that is possible) into the future, towards what it may feel like, when she finishes her degree:

right at the beginning she brought along the doctoral hat and she made us each put it on and take photos and she said when things are getting a bit tough you might like to look at these photos and I just laughed, I thought oh what a joke, you know, but it is actually quite a nice thing to imagine yourself sometimes at the end which ties in with commitment. And sometimes you need to have something there that pushes you along and that’s, I guess it goes hand in hand with commitment I suppose. (distance, Participant 3).

The symbolism of objects that represent their connection to the university can also be used to express their identity as members of the university community. Wearing the regalia at public events is one example of how students feel they can be identified as someone who belongs to the university, as one who has ‘earned’ the right to display those clothes at public celebrations. Participant 3 expresses the power of materials that represent her sense of belonging to the university community:

I must confess that when I graduate I think there will be a tremendous sense of belonging and identity wearing the robe, a typical example is actually happening in a week or so, I am going to a graduation ceremony here in (city). I am on a, a board at the, well it is at the (name) tertiary institute here in (city) so as one of the board members I am going along. We wear our academic regalia and I felt, I should have mentioned that actually because I was just filling out a form this morning confirming what I was going to wear and I always feel proud to say that I am from [this] University and to wear the regalia but that sort of identifies me as a, I suppose a professional academic in a way that I have earned the right to wear those clothes, even though it’s my masters at the moment, it will be, maybe next year it might be my doctoral robes. (distance, Participant 3).

Connected to this sense of being part of a community is the need for networking amongst the members of the academic community. The materiality of spaces where doctoral students feel connected to other academics (Barnacle and Mewburn 2010) was alluded to at several passages in the interviews, and through images and audio captured in the postcards created by on-campus students. These passages highlight the importance of the staff room as a place to get closer to others to have interesting conversations. Participant 1 comments on the staff room as a space to share ideas with like-minded others:

being on-campus allows me to have access to supervisors and help. … We connect [at the staff room] with academics, they have seminars, which is good. … It is very important … because networking is part of your learning, you’ve got to do that, especially in academia, because they are all in different functional departments, different intelligences, you’ve got to share those ideas, and you learn from others too. (on-campus 1, Participant 1).

**Fig. 5:**
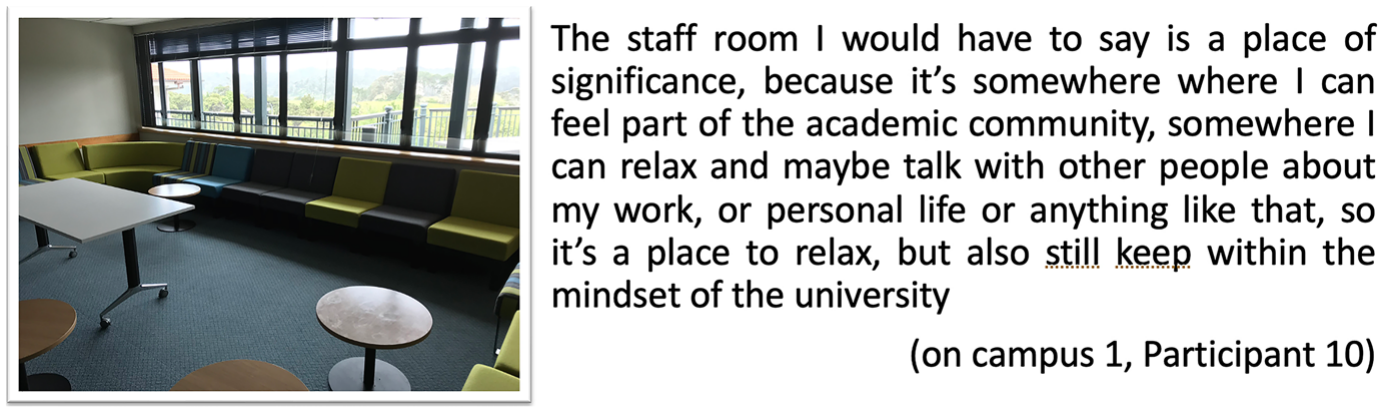
Postcard staff room, on-campus 1, Participant 10

Digital postcards also reflected similar sentiment, displaying pictures of the staff room to express their value of the types of desirable interactions that are possible in this place, to mediate conversations and to feel part of an academic community, to connect and develop networks (Fig. 5).

**Fig. 6:**
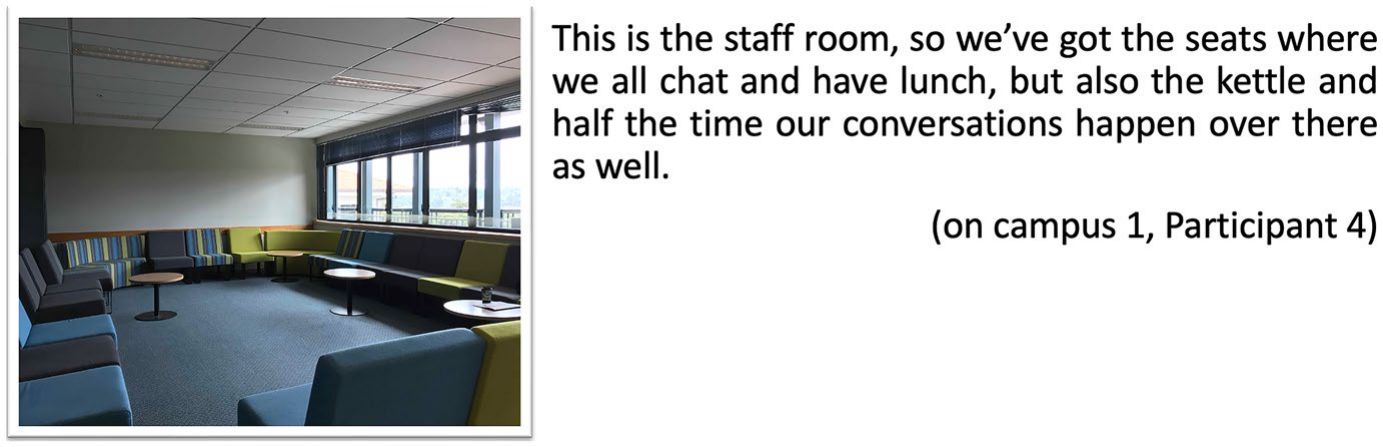
Postcard staff room, on-campus 1, Participant 4

A postcard by Participant 4 also brings the same representation with similar references — the staff room as a space to have conversations (Fig. 6).

Virtual spaces also contribute to a perceived value of the institution, and to participants’ identity. For example, Participant 5 alludes to the symbolic nature of the institution, speaking about her relationship with ‘this University’ as mediated through the digital, via the many online conversations and mentoring by staff. This relationship is perceived as deeply shaping her life and work, and her identity as a professional:

when I think about my relationship with the University and the University staff you know I look over the span of a number of years but I must say that in all the, so the conversations I have had with my lecturers and just the support I got from them and the, the, the values and the theoretical approaches I learned from them have shaped who I am as a person and as a, as a practitioner and has guided me through, through life and work. (distance, Participant 5).

This perceived value can also perhaps be extrapolated to other digital forms of identity, such as when students have opportunities to interact or create profiles on Faculty webpages, through the use of an institutional email or access to staff information, ethics platforms as already alluded to before.

### Theme 3: Nature Contributing to Reflections

Students’ symbolic relationships to a physical campus have also included a range of informal representations, for example, as they see images or objects that they associate with the physical campus, a sense of connection is extended through the digital realm. Participant 3, for example, is a distance student who refers to being reminded about an environment to ‘grow intellectually’, whilst looking at images uploaded by a friend on Facebook. She speaks of a feeling of connection to buildings, but also to the nature in and around the university physical campus.

(Campus 2) is just a beautiful environment to learn and to grow intellectually, it is just lovely and I never tired of it … (a friend recently) did a number of photographs around the campus of flowers and buildings and oh you name it, out of the way places and she put them up on Facebook and it was just lovely, it was, and a lot of the time I think I know that place, I know that place and it was just so nice and especially when we live here it was nice to be reminded of [Campus 2]. (distance, Participant 3).

**Fig. 7:**
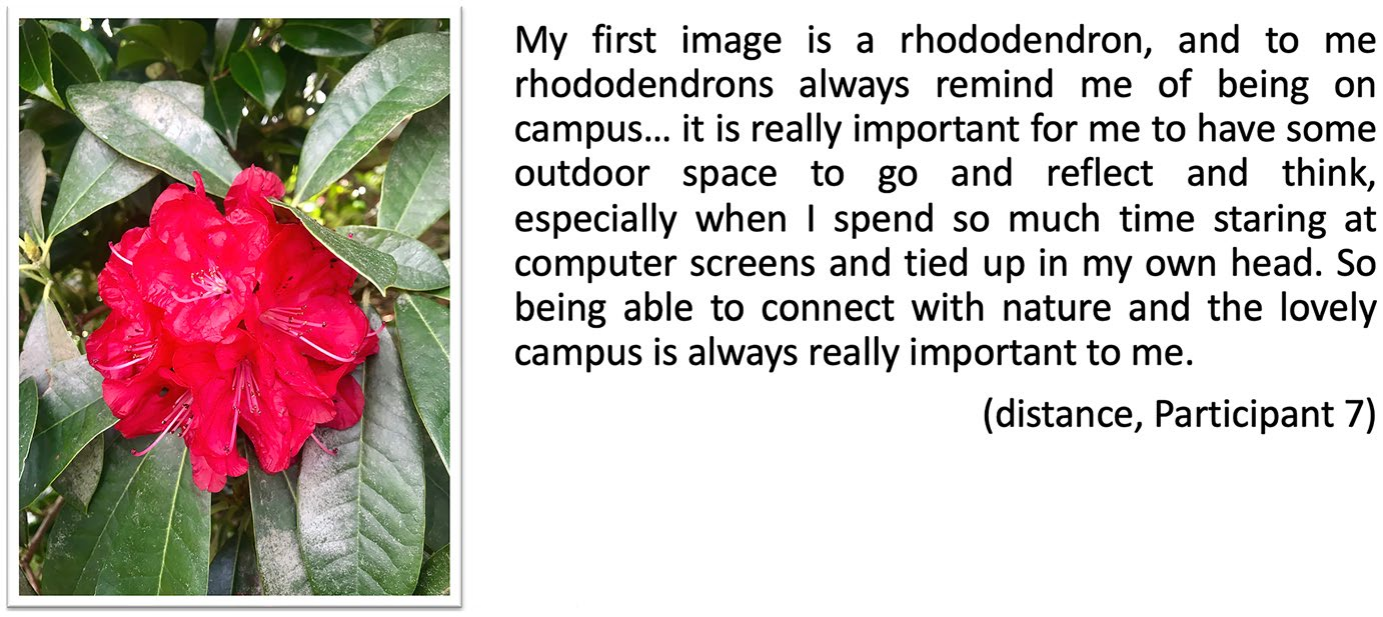
Postcard flower, distance, Participant 7

The connection to nature invoked by Participant 3 also appears in the digital stories of other distance students. For example, Participant 7 uses the image of a rhododendron taken elsewhere and links the flower to her representation of the physical campus (Fig. 7). She talks about the importance of the outdoors as experiences to reflect and think, and to have a break from screens.

**Fig. 8:**
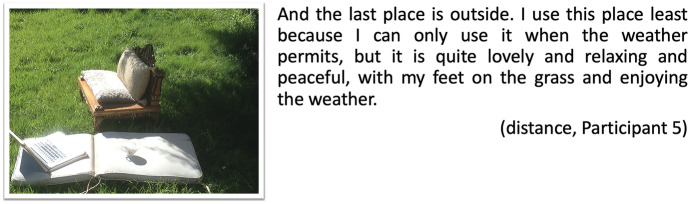
Postcard garden, distance, Participant 5

**Fig. 9:**
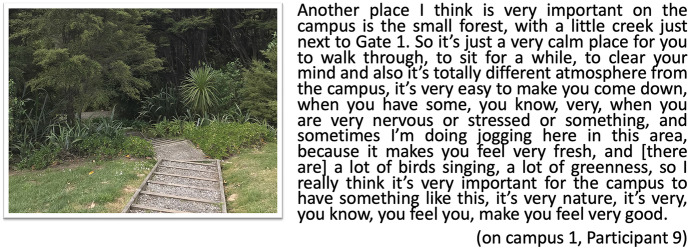
Postcard creek, on-campus 1, participant 9

A digital postcard created by Participant 5 also references to the importance of nature around (Fig. 8). Nature brings a feeling of relaxation and warmth as a peaceful place for learning:

Similarly, digital stories by on-campus students also mention nature (Fig. 9), for example when Participant 9 touches on the importance of nature around the campus, as a place that is valued and cherished to help ‘clear the mind’:

## Discussion: Places for Learning and the Materiality of the University

As the above themes illustrate, a wide range of learning places exist, and these go far beyond lecture theatres and libraries in a physical setting. Boundaries between material and digital, formal and informal learning are blurred, and learning activity is connected to multiple spaces, including those described as being at the in-between (Boys 2011; Gourlay and Oliver 2018; Wardak et al. 2022). Such ubiquity of learning has deep implications for the many ways people might experience and connect to places for learning in and around universities (Lamb et al. 2022).

The interviews and digital postcards created by on-campus and distance students described their attachment to places and the significance of material and digital representations connected to their experiences and identities as doctoral students. They reported experiences related to the emotional, symbolic and affective dimensions of their connections to places and material things (Altman and Low 1992; Rishbeth and Powell 2013). They also reflected aspects of themselves as a ‘person’ — telling us a little bit about who is attached and why. They spoke about ‘places’ and materials, through accounts that explain what people are attached to and why. They also hinted to the ‘process’, revealing how a place might have become meaningful to someone and their impact on individuals (Scannell and Gifford 2010). Students not only stressed the importance of the materials and spaces that mediate their social interactions, but also spoke about how these allow them to feel connected to a community. The doctoral students who participated in the study seemed acutely aware that their participation in the practices of the university community is shaping their identities as emerging scholars (Wenger et al. 2011). Spaces and connections that are perhaps more unusual were also mentioned, like those mediated by nature, such as a garden, a creek, a flower and places that help them to unwind and reflect on their learning experiences.

On-campus students referred to their allocated Ph.D. space as offering a sense of purpose, through having a physical location to study as well as by reinforcing their role/identity as members of the university community. Being physically on campus positioned these students alongside like-minded others. As Edwards and Usher (2008) suggest, this interconnection of location, space, role and identity is highly relevant in helping students feel they belong within an institution. This sense of belonging supports the development of their identity as future academics, researchers, professionals and in construing and reflecting a certain status, which allows students to negotiate the ambiguous boundaries of the doctoral candidature. While the role of locality is strongly bounded to the narrative of the on-campus students, through the physical proximity to peers and academics, as well as allocated spaces, a sense of belonging is also alluded to in the narratives of distance students in the study, suggesting that these students too are forging new proximities (Bayne et al. 2014). Distance students referred to materials when mentioning the proud feeling of wearing the university regalia to represent their community, or to a rhododendron that symbolically reminded them of the university as a place to grow intellectually. As such, the qualities of the materials (e.g. university regalia) and the symbolism of an object (rhododendron) allow these students to feel connected, to situate themselves as part of the university community.

While this study focused on the experiences of doctoral students in a New Zealand university, the experiences reported here are perhaps not too dissimilar to the symbolic and material significance of the physical campus reflected by doctoral students at other universities. Indeed, similar accounts have appeared in social media recently, for example in posts by doctoral graduates during the Covid-19 lockdown. An interesting example comes from The University of Sydney and is related to one of the long-standing traditions of graduation ceremonies at this university, which involves students taking a picture near a beautiful Jacaranda tree. The tree was planted in the lush lawns of the Quadrangle Building many years ago and had been the backdrop for thousands of graduations over its 88-year lifetime, until 2016 when the Jacaranda died. In 2014, as it was noticed that the tree was nearing the end of its life, the university organized for a specialist grower to take cuttings, which were then grafted onto the base of other Jacarandas to produce two clones so that The University of Sydney could replace the original Jacaranda tree with a genetically identical one (The University of Sydney 2016). The symbolic importance of the Jacaranda tree for this community cannot be underestimated. The Jacaranda’s symbolism, and its significance, materialized recently via social media, as a postgraduate student who had recently graduated — posted her image smiling and holding her degree on the lawns of the Quadrangle Building. The text accompanying the image explained that having finished her degree during the Covid-19 scenario, she missed out on a physical graduation ceremony. So, this doctoral graduate attended the campus with her family on a weekend, to get a picture of herself holding her degree at the physical place that holds significance to her learning journey: against the backdrop of the Jacaranda and the place that has occupied a symbolic status for graduation ceremonies at that university for almost a century. The symbolism that a physical campus holds is nicely illustrated in this story, and its interconnection with the digital is not to be missed — as the student attends the physical campus to ‘truly’ finalize her learning journey, and share her achievement with her network of professional colleagues and friends through the digital.

Overall, many of the stories by doctoral students allude to the materiality of the physical campus and to representations of their experiences of being ‘at the university’. Digital and material elements contribute to their learning in subtle ways, by positioning, allocating and bringing opportunities to learn and to feel a sense of connection, including those afforded through the serendipity of encounters with others. Both on-campus and distance students brought stories that reflected a complex network of elements, which includes the spatial arrangements of materials and the built environment and their influence on learning experiences, knowledge construction and identity formation (Barnacle and Mewburn 2010).

## Conclusion

Doctoral students’ experiences involve transformations, as they take on a new identity as an academic or professional in a particular field. Social connections are important for students’ physical and emotional well-being, encouraging progress and career development (Peltonen et al. 2017), as people are more likely to thrive when they feel connected, when they feel they belong to a community (Mantai 2019). Students’ experiences of places for learning and the materiality of things that surround doctoral students at a multi-campus university suggested that the proximity to peers and academics and the opportunities to informally interact with others were highly valued by on-campus students. Distance students do not often come to campus and only seldom experience a sense of physical proximity to peers and other academics. Nevertheless, these students express that they feel connected when seeing digital representations via social media, when dressing in the university regalia, when attending workshops or when meeting with their supervisors via digital conference calls. All these experiences were perceived as contributing to their feelings of connections to the university community. The next phase of this study considers ways of sharing experiences of placemaking and belonging through a digital platform, as a way of bringing people together within a community of learners.
